# The complete mitochondrial genome of small wax moth, *Achroia grisella* (Pyralidae: Galleriinae)

**DOI:** 10.1080/23802359.2022.2068984

**Published:** 2022-05-03

**Authors:** Yangyang Liu, Guoyong Li, Jiankun Long

**Affiliations:** a Guizhou Provincial Key Laboratory for Rare Animal and Economic Insects of Mountainous Region, Guiyang University, Guiyang, People’s Republic of China; b Institute of Entomology and Special Key Laboratory for Development and Utilization of Insect Resources of Guizhou, Guizhou University, Guiyang, People’s Republic of China

**Keywords:** *Achroia grisella*, mitogenome, homoeology, phylogenetic analysis

## Abstract

We sequenced and annotated the complete mitochondrial genome of *Achroia grisella* (Fabricius, 1794), which was 15,368 bp, encoding 13 protein-coding genes (PCGs), 22 tRNA (tRNA) genes, 2 ribosomal RNA (rRNA) genes, and a control region; having a base composition of A (38.7%), C (12.3%), G (7.5%), T (41.5%), G + C (19.8%), and A + T (71.2%); except for the ND1, which was initiated by the GAT codon, the other 12 PCGs were initiated by the ATN (ATT, ATA, and ATG) codon. The nine PCGs terminated with the typical TAA stop codon, whereas the remaining four genes had incomplete stop codons, which were single T. In addition, the phylogenetic relationships based on nucleotide sequences of 13 PCGs using Bayesian inference (BI) and maximum likelihood (ML) methods showed a close relationship between *Achroia grisella* and *Galleria mellonella*.

The small wax moth, *Achroia grisella* Fabricius, belongs to the Lepidoptera family Pyralidae genus *Galleria* Fabricius insects, and both *Achroia grisella* larvae and *Galleria mellonella* Linne larvae are collectively referred to as nests pest (Wen 2008). The damage of *Galleria mellonella* to honeybees commonly occurs in bee-keeping areas of South China. *A. grisella* lives at the bottom of the hive and feeds on wax chips dropped from the bottom of the hive, which can also destroy the unpreserved nest spleen. (Seeley 2009; Zhang et al. 2019; Mahgoub et al. 2020). The larva of the great wax moth feeds on the nest spleen of bees and causes a large number of capped pupae to die or the bee colony to give up the nest, which is seriously harmful (Kwadha et al. 2017). Therefore, less attention has been paid to the small wax moth, and there is some confusion in morphological identification between *A. grisella* and *G. mellonella*, which are not easy to identify (Zhang et al. 2019). In this study, we focused on the mitochondrial genome of *A. grisella* in an attempt to analyze the phylogenetic relationship and evolutionary relationship of *A. grisella*, which has important biological significance for the species identification and genetic background of *Achroia grisella* from the molecular biological level.

The specimens used in this study were collected from Yongai Village, Shangtang Town, Kaili City, Guizhou Province (Miaojiang bee breeding base, Huangping County) (107.69E, 26.91 N) and were deposited in the Guizhou Provincial key laboratory for rare animal and economic insects of mountainous region (Ms. Liu, tadouliuyy@126.com) (Certificating number: GY2021090101). Among them, the common method to collect insect samples is to immerse the insects in alcohol immediately after they are captured as a sample for DNA extraction. This does not torture insects and is not unethical.

A sample’s total genomic DNA was extracted from each individual using the TIANGEN Genomic DNA Extraction Kit (TIANGEN Co., Beijing, China) according to the manufacturer's protocol, sequencing libraries with an average length of 350 bp were generated using the TruSeq Nano DNA HT Sample Preparation Kit (Illumina USA), and then the libraries were sequenced on the Illumina Novaseq 6000 platform. After quality checking of the obtained sequences, raw sequence reads were edited using the NGS QC toolkit (Patel and Jain 2012), clean data were *de novo* assembled by Mitoz v. 2.3 software (Meng et al. 2019). Finally, annotation of the mitochondrial genomes was performed using Geneious R10 (Biomatters, Auckland, New Zealand), with BLAST against and related species from GenBank as the reference (Altschul et al. 1990; Kearse et al. 2012), and submitted to GenBank (accession number: OM203125) (https://www.ncbi.nlm.nih.gov/.) and SRA (accession number: PRJNA792436) (https://www.ncbi.nlm.nih.gov/bioproject/PRJNA792436).

Twenty-two mitogenomic sequences (downloaded from GenBank) were used in the phylogenetic analysis, and 13 coding protein genes of all 21 insect species and *Achroia grisella* were extracted using Geneious R10, with *Loxostege sticticalis* and *Ostrinia furnacalis* as outgroups. We then used Clustal X 1.83 (Thompson et al. 1997) and SequenceMatrix 1.8 using default settings (Vaidya et al. 2011), and 13 protein-coding genes were concatenated as a dataset. Nucleotide matrices were used for phylogenetic analysis using two approaches: Bayesian inference (Ronquist et al. 2012) and Maximum likelihood (Nguyen et al. 2015). The best-fit models (GTR + I + G and GTR + F + I + G4) for the nucleotide sequence were selected using the Akaike Information Criterion (AIC) in jModeltest 2.1.10 and IQ-TREE 1.6.2, respectively (Darriba et al. 2012; Nguyen et al. 2015). The resulting phylogenetic tree is edited and visualized in FigTree 1.4.0 (Mousavi et al. 2014) (Figure 1).

**Figure 1.**
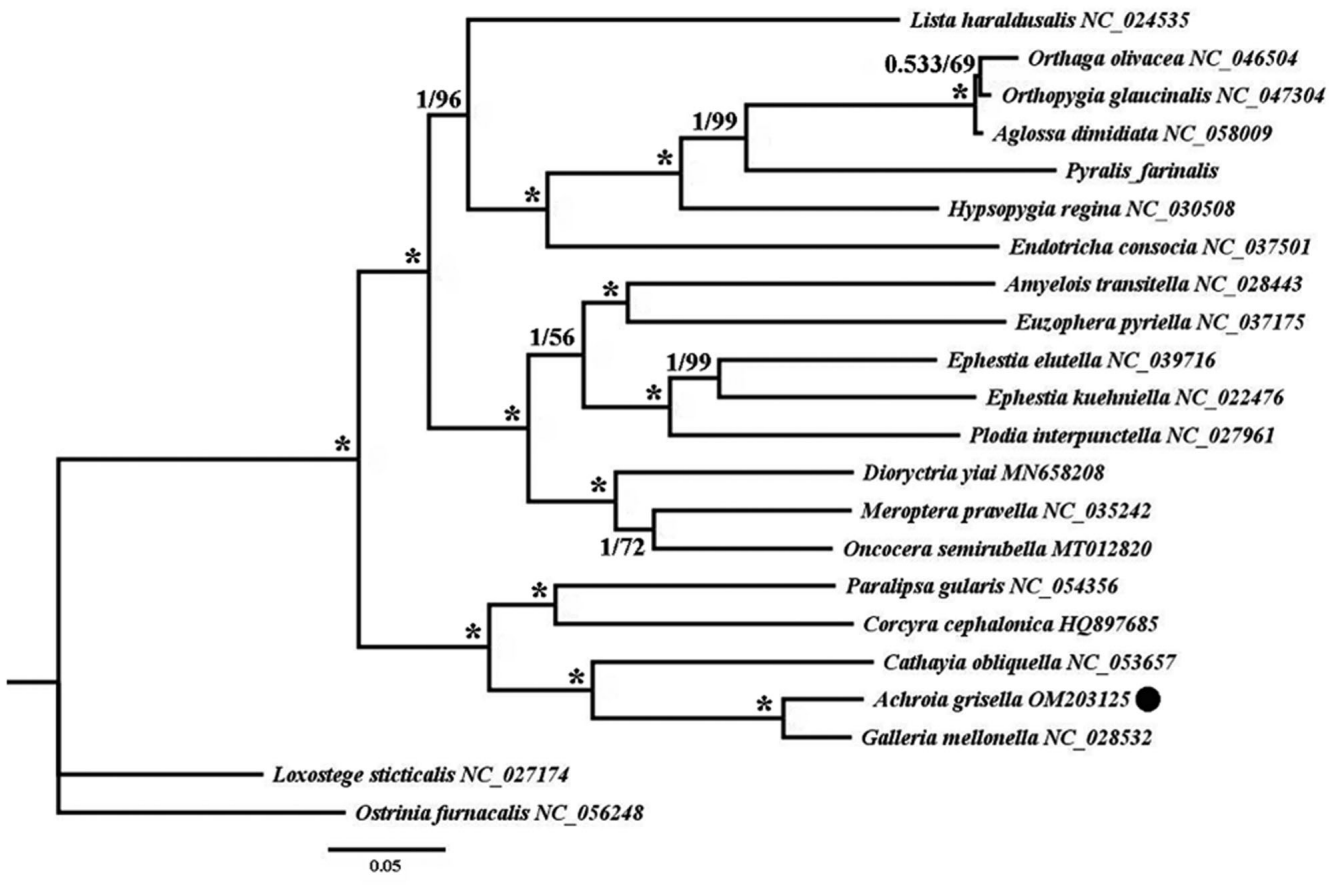
The phylogenetic trees of *Achroia grisella* were inferred from the amino acid sequences of the 13 PCGs in mitogenome of 22 species from using Bayesian inferences (BI) and maximum likelihood (ML) methods. Numbers on branches indicate BI posterior probability (PP, left) and ML bootstrap support (BS, right), respectively. An asterisk denotes PP =1 and BS = 100. The black dot indicates mitogenome of *Achroia grisella* newly determined in this study.

The complete mitochondrial genome sequence of *A. grisella* (GenBank: OM203125) is 15,368 bp, with base compositions of A = 38.7%, C = 12.3%, G = 7.5%, and T = 41.5%. Our sequences are similar to those of other metazoans, which contain 13 protein-coding genes, 22 tRNA genes, 2 rRNA genes, and a non-coding AT-rich region. Except for the *ND1*, which is initiated by a particular GAT codon, the other 12 PCGs are all initiated by ATN (ATT, ATA, and ATG) codons. Of these, three PCGs (*COX3*, *ND4L*, and *ATP6*) started with ATG, seven PCGs (*COX1*, *COX2*, *ND2*, *ND3*, *ND4*, *ND5*, and *ATP8*) started with ATT, one PCG (*CYTB*) started with ATA, and one PCG (*ND6*) started with ATC; in addition, nine PCGs (*ND2*, *ND3*, *ATP8*, *ATP6*, *ND4L*, *ND5*, *ND6*, *COX3*, and *CYTB*) terminated with TAA codons and four PCGs (*ND1*, *ND4*, *COX1*, and *COX2*) had incomplete stop codons, which were single T.

Phylogenetic relationship analysis (Figure 1) showed that *A. grisella* belongs to the genus Galleriinae and has the highest homology and closest genetic relationship with *Galleria mellonella*, which is consistent with the results of phylogenetic analysis of the small wax moth based on the cytochrome c oxidase subunit I gene by Zhang et al. (2019). In addition, it was found that *A. grisella* was clustered with other Pyralidae insects. Therefore, according to the analysis, we could further reflect the evolutionary relationship of *A. grisella* and clarified that the complete mitochondrial genome sequence of *A. grisella* was the evolution of the Pyralidae, thus providing a theoretical basis for exploring the genetic diversity and phylogenetic study of the main enemy of the colony.

## Authors’ contributions

Yangyang Liu, analyzed the data, performed the experiments, involved in certain tools for analysis, prepared figure, drafted of the paper, and approved the final draft.

Guoyong Li, collected and analyzed data, consulted the relevant literature, and uploaded the analysis data.

Jiankun Long, contributed reagents/materials, involved in conception and design of the work, and approved and published the final draft.

All authors agree to be accountable for all aspects of the work.

## Data Availability

The data that support the findings of this study are openly available at NCBI database. The GenBank Accession No. (reference number) is OM203125 (https://www.ncbi.nlm.nih.gov/.). The associated BioProject, SRA, and Bio-Sample numbers are PRJNA792436, SRS11402858, and SAMN24425024, respectively, and all of the accession numbers are activated.
